# Revisiting Ayurveda for Pediatric Oral Health: A Randomized Controlled Trial Evaluating the Effects of Coconut Oil and Castor Oil Pulling on Plaque Accumulation, Gingival Health, and Salivary Bacterial Load in Children

**DOI:** 10.7759/cureus.115573

**Published:** 2026-09-01

**Authors:** Prachi Deshmukh, Deepika Jauhari, Puneet Gupta, Rachana Mishra, Pratiksha Patil, Mrinal Bhatia

**Affiliations:** 1 Pediatric Dentistry, Daswani Dental College and Research Centre, Kota, IND; 2 Pediatric and Preventive Dentistry, Daswani Dental College and Research Centre, Kota, IND; 3 Conservative Dentistry and Endodontics, Daswani Dental College and Research Centre, Kota, IND

**Keywords:** ayurveda, castor oil, coconut oil, gingival index, oil pulling, pediatric oral health, plaque index

## Abstract

Background and aim

Oil pulling (OP) has been studied as an adjunct to routine oral hygiene, but pediatric evidence - especially for castor oil - is limited.

The registered primary outcome broadly covered gingival health and bacterial flora at two weeks; no secondary outcome was registered. This report compared those components after virgin coconut oil (VCO), virgin castor oil, or distilled-water rinsing. Plaque Index and the direct VCO-versus-castor comparison were exploratory.

Materials and methods

This exploratory randomized controlled trial enrolled 70 children, of whom 51 met the eligibility criteria and were allocated equally to VCO, castor-oil, or distilled-water groups (n = 17 each). Participants performed the assigned intervention once daily for two weeks. The Turesky modification of the Quigley-Hein Plaque Index, the Löe-Silness Gingival Index, and salivary MacConkey-culturable bacterial counts were assessed at baseline (T0) and after two weeks (T2). Intergroup values were compared using one-way analysis of variance (ANOVA) followed by Tukey HSD tests (p < 0.05).

Results

Forty-five children (n = 15 per group) completed the study. Both oil-pulling groups showed statistically significant reductions in Plaque Index, Gingival Index, and bacterial load from baseline to the two-week follow-up (p < 0.05). At postoperative evaluation, Group B (castor oil) demonstrated the greatest reduction in Plaque Index (3.90 ± 0.46 to 2.50 ± 0.38) and Gingival Index (2.00 ± 0.32 to 0.90 ± 0.28). Bacterial counts decreased markedly in both Groups A and B (from approximately 6.75 and 6.65 to 5.70 and 5.65 × 10³ CFU/mL, respectively), whereas Group C showed negligible change (6.55 to 6.50 × 10³ CFU/mL). Intergroup comparisons confirmed significantly better outcomes in both intervention groups relative to the control.

Conclusion

Over two weeks, VCO and castor-OP were associated with lower mean plaque and gingival-index scores and lower salivary MacConkey-culturable bacterial counts than distilled-water rinsing. The small sample, baseline imbalances, short follow-up, and restricted microbiological method limited causal and clinical interpretation. Larger trials using baseline-adjusted analyses and oral-microbiome-specific methods are required before routine paediatric use can be recommended.

## Introduction

Oil pulling (OP) is an ancient practice of oral cleansing that originated in India [1-3]. This practice consists of swishing a tablespoon of edible oil in the mouth cavity for a specified period of time before expectorating it. It is said that OP "pulls" away toxins and harmful organisms from the mouth. Traditionally, sesame oil and sunflower oil are used for OP. In recent years, however, due to a variety of advantages that are believed to result from OP, this technique has gained increasing interest all over the world [4-8].

The clinical efficacy of OP as an oral hygiene intervention needs to be determined or assessed in terms of standardized and reproducible oral health indices, e.g., the decayed, missing, and filled teeth (DMFT) index, originally described by Klein, Palmer, and Knutson and later adopted by World Health Organization oral health survey methods [9]. Plaque levels can be measured using the Quigley-Hein Plaque Index [10] and the modified Quigley-Hein Plaque Index proposed by Turesky [11]. The Löe and Silness Gingival Index is used for measuring levels of gingival inflammation [12-19]. These indices provide widely accepted and objective ways to measure and compare changes in plaque and gingiva levels following OP. Several systematic reviews and meta-analyses conducted recently have revealed some level of evidence that OP may help in reducing the amount of plaque in the mouth cavity and in decreasing gingival inflammation, similarly to the use of chlorhexidine mouth rinse [2-4,20-29].

Virgin coconut oil (VCO) is the most studied type of oil among all types used in OP and has proved its efficacy in reducing gingivitis induced by plaque [19,30]. Peedikayil et al. and Kandaswamy et al. observed that coconut OP resulted in a reduction of Plaque Index and Gingival Index scores in children aged 8-12 years [21,31]. Moreover, Kaushik et al. [29] noted that VCO was comparable in effectiveness to chlorhexidine mouth rinse in reducing levels of salivary *Streptococcus mutans*.

Another type of oil that has been studied less often than VCO but still holds certain antibacterial and anti-inflammatory qualities due to ricinoleic acid, a compound that constitutes castor oil (*Ricinus communis*). Due to its wound-healing properties, castor oil was used in traditional medicine. However, there is a need to evaluate this compound as a tool for maintaining oral health because of its potential in preventing biofilm formation [17].

Most of the publications in recent years concern only adults and are mainly based on using sesame oil or VCO, while evidence about the comparative efficiency of different oils used for OP in children is very scarce [18,20,21]. No clinical trial so far has been conducted to compare the effects of VCO and castor oil in children. As OP can be considered a promising way to achieve better oral hygiene, especially in developing countries where there is a lack of access to professional oral care services, further research is needed to determine which oil is preferable for OP in children. Therefore, this study evaluates the effects of OP with VCO and castor oil on plaque formation, gingival inflammation, and bacterial levels in saliva in children aged 9-14 years.

## Materials and methods

Study design and ethical approval

This study was designed as a three-arm, parallel-group, randomized controlled trial. Ethical approval was obtained from the Institutional Ethics Committee of Daswani Dental College and Research Center, Kota, India, on 16 February 2026 (approval no. DDC/Acad/2025-26/2920). The study was conducted in accordance with the principles of the Declaration of Helsinki. Written informed consent was obtained from the parents or legal guardians of all participating children after an explanation of the protocol, objectives, and potential risks. The trial was prospectively registered with the Clinical Trials Registry-India (CTRI) (CTRI/2026/03/105227).

Study population

A total of 70 children were assessed for eligibility. The inclusion criteria were as follows: (i) children aged 9-14 years; and (ii) a DMFT score of 1-2, with an otherwise good general health status [9]. The exclusion criteria comprised: (i) children who had received topical fluoride treatment within the preceding two weeks or systemic antibiotic therapy within the past three months; (ii) children with systemic illnesses; (iii) those using any other mouth rinse; (iv) those undergoing orthodontic treatment; and (v) those with deleterious or parafunctional oral habits. Following the application of these criteria, 19 children were excluded, and the remaining 51 were enrolled and randomized.

Randomization and group allocation

The 51 eligible participants were randomly allocated in equal numbers (n = 17 per group) to one of three study arms: Group A, OP with cold-pressed VCO; Group B, OP with cold-pressed virgin castor oil; and Group C, rinsing with distilled water (control group). During the course of the study, two participants from each group were lost to follow-up (total dropout = 6), yielding a final analytical sample of 45 children (n = 15 per group).

The sample size was estimated for a one-way analysis of variance (ANOVA) comparing three independent groups. Assuming a Cohen’s effect size of f = 0.48, a significance level of 5%, statistical power of 80%, and equal allocation, the minimum required final sample was 45 participants. Accordingly, 15 participants were included in each of the coconut-oil, castor-oil, and distilled-water groups. The patient flow through the study is depicted in Figure 1.

**Figure 1:**
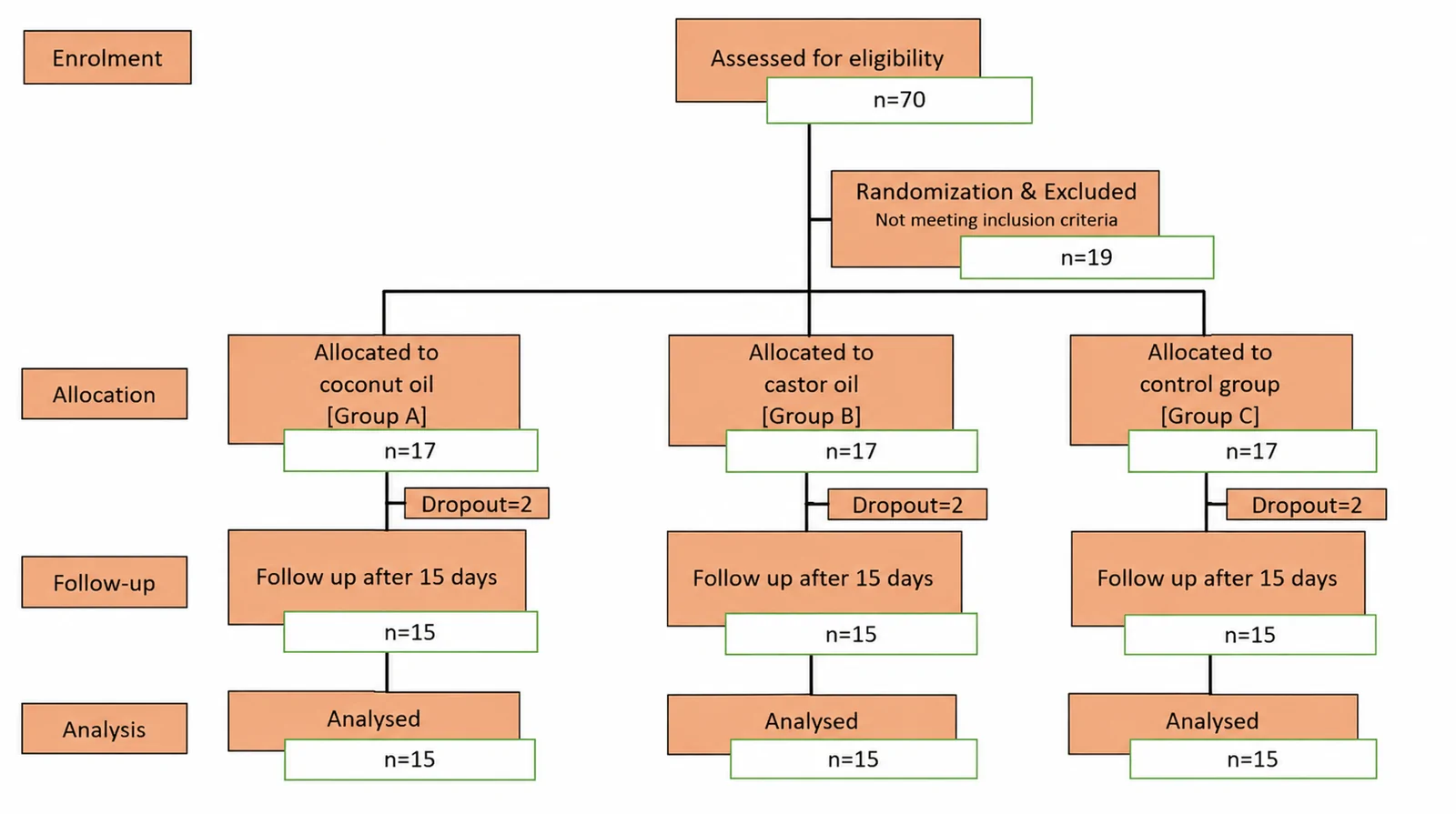
Patient selection framework for the study

Intervention protocol

All participants were instructed to perform the assigned intervention once daily, preferably in the morning on an empty stomach, for a period of two weeks. Participants were clearly informed to take adequate precautions not to swallow the liquid during the demonstration of the procedure. The oil-pulling protocol for Groups A and B was as follows: the child was asked to take one tablespoon (approximately 10-15 mL) of the assigned oil into the mouth, swish it gently around the oral cavity for 10-15 minutes, and then expectorate the oil into a disposable container. After expectoration, the mouth was rinsed with warm water, followed by regular tooth brushing. Participants in Group C were instructed to rinse their mouth with an equivalent volume of distilled water under the same conditions [4]. Compliance was monitored through a dedicated WhatsApp group, which facilitated daily reminders, real-time communication, and instant updates between the investigators and the participants’ guardians. Compliance was supported through a dedicated WhatsApp group that provided daily reminders and communication with guardians. However, the number of completed sessions was not independently verified or quantified, and adherence could therefore not be formally compared among groups.

Clinical assessment

The clinical examinations were carried out at two time points. The first time point, designated T1, was an evaluation performed immediately after the first intervention session. The second time point, designated T2, was an evaluation performed after 15 days of daily intervention. Two validated indices were used as instruments for clinical evaluation. The first was the Turesky modification of the Quigley-Hein Plaque Index [10,11]. This index was used to measure supragingival plaque accumulation and ranged from 0, indicating no plaque, to 5, indicating that plaque covered two-thirds or more of the tooth surface. The other was the Löe and Silness Gingival Index [12], which was used to evaluate the degree of gingivitis. Figure 2 shows the preoperative view of a participant in the VCO group before the commencement of OP with VCO.

**Figure 2:**
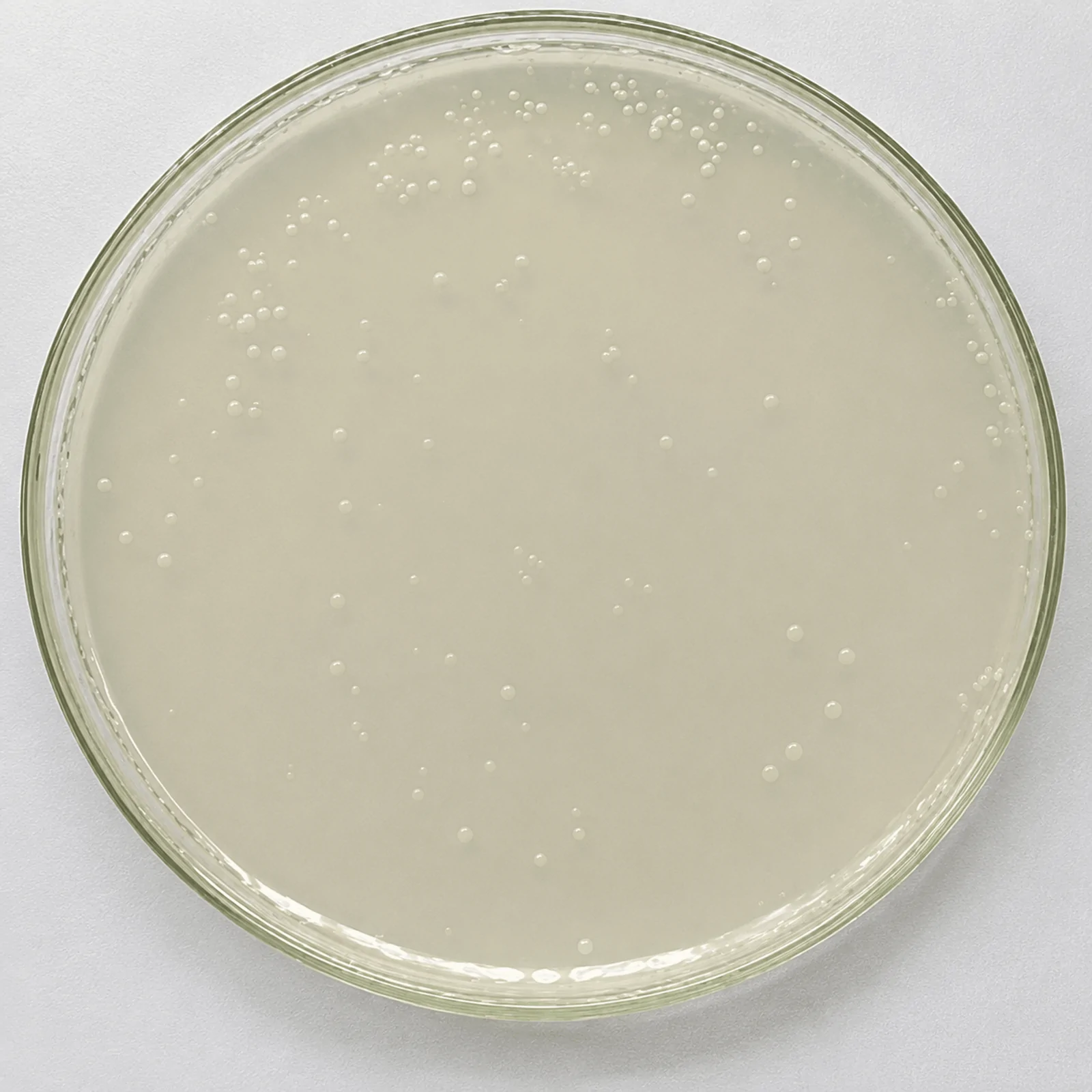
Preoperative oil pulling using virgin coconut oil

Figure 3 depicts the postoperative appearance of participants in the VCO group two weeks after completing OP with VCO. The figure illustrates the condition of the participants after completion of the experiment and reflects the post-intervention status of the coconut oil group.

**Figure 3:**
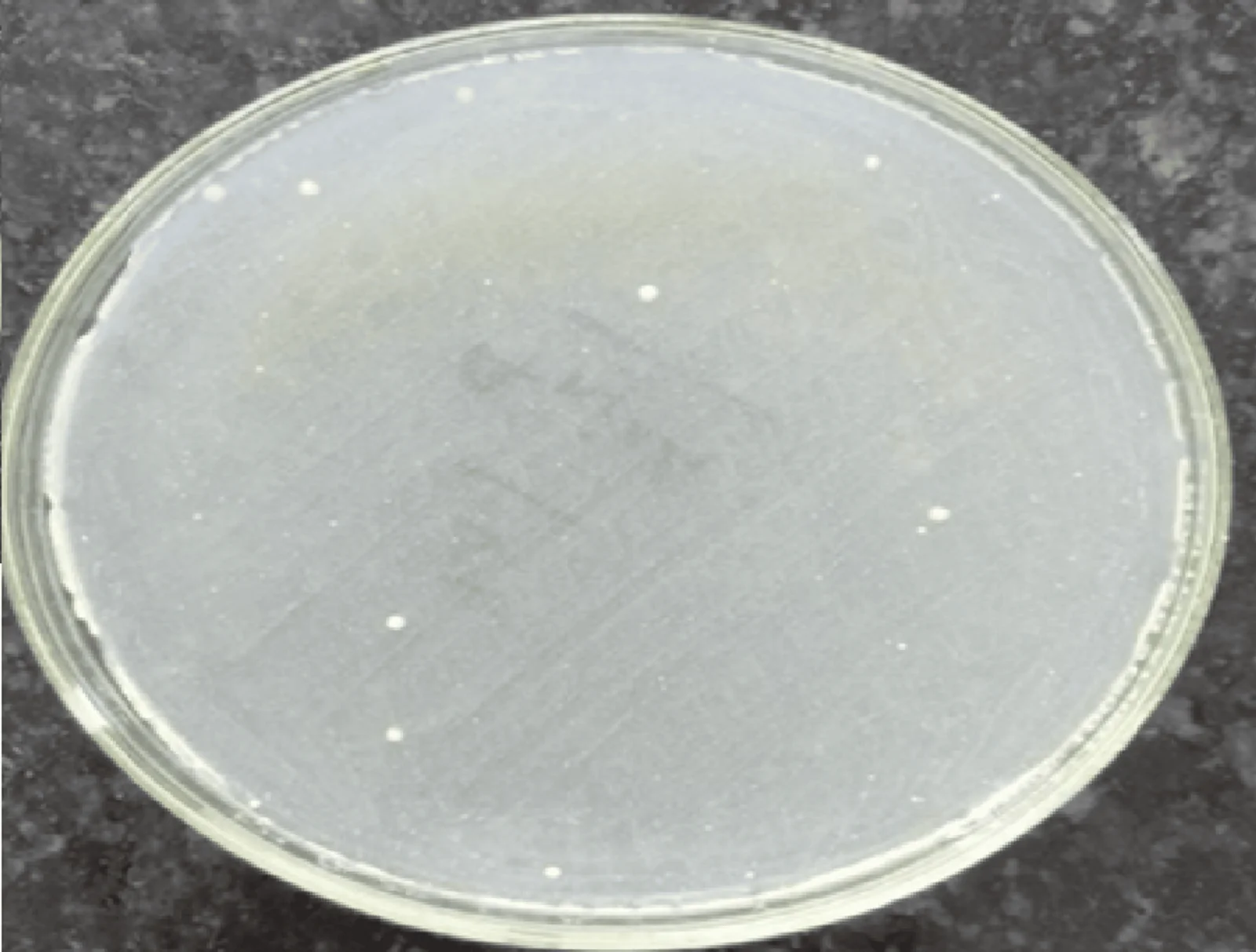
Two weeks postoperative oil pulling using virgin coconut oil

Figure 4 depicts the preoperative image of a participant from the virgin castor oil group before the intervention with virgin castor oil. It represents the pre-intervention status, which was later compared with the post-intervention status depicted in the following figure.

**Figure 4:**
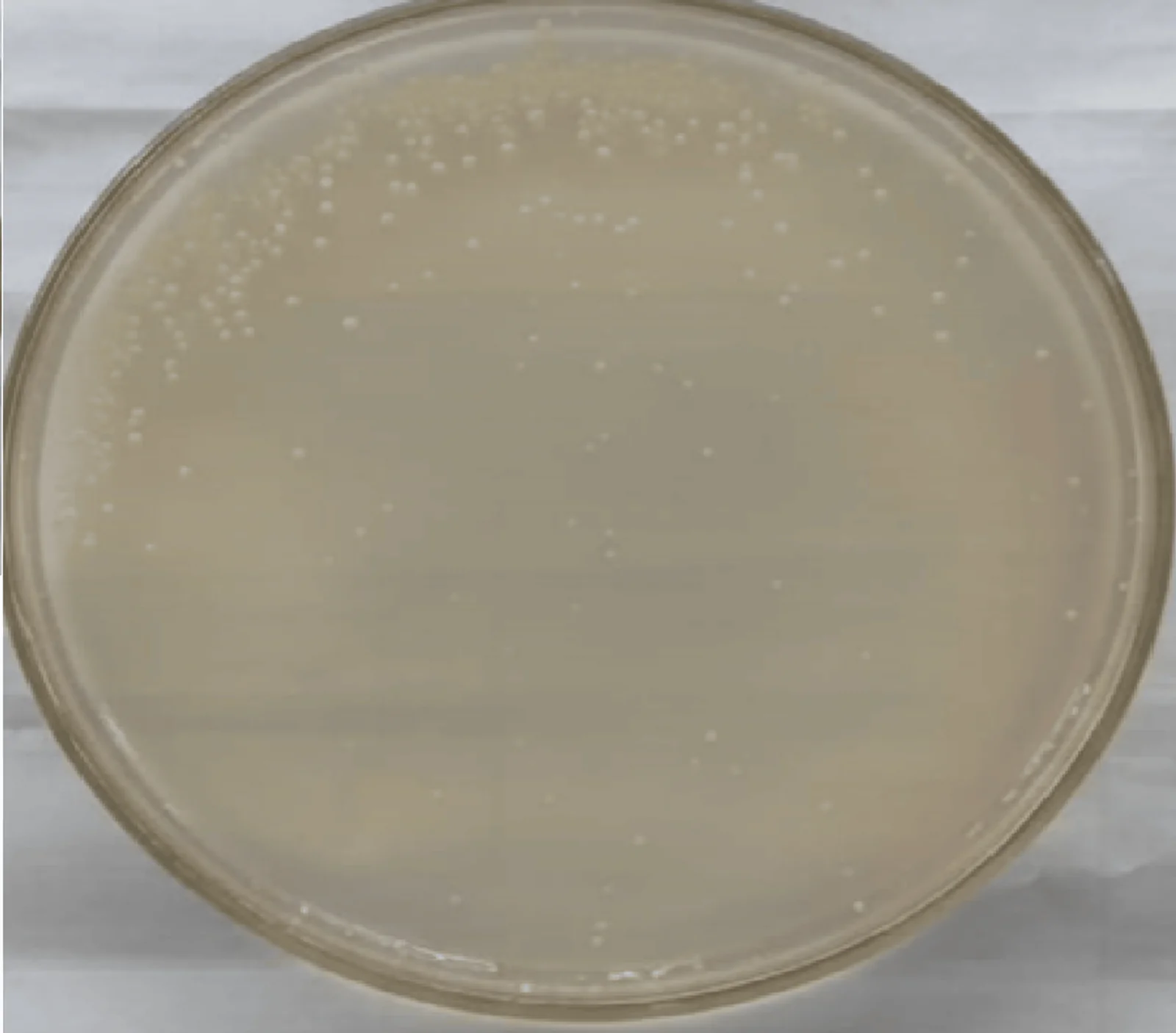
Preoperative oil pulling using virgin castor oil

Figure 5 depicts the postoperative image of participants in the virgin castor oil group two weeks after completing OP with virgin castor oil. It illustrates the post-intervention status of this group and allows visual comparison with the corresponding pre-intervention findings shown in Figure 4.

**Figure 5:**
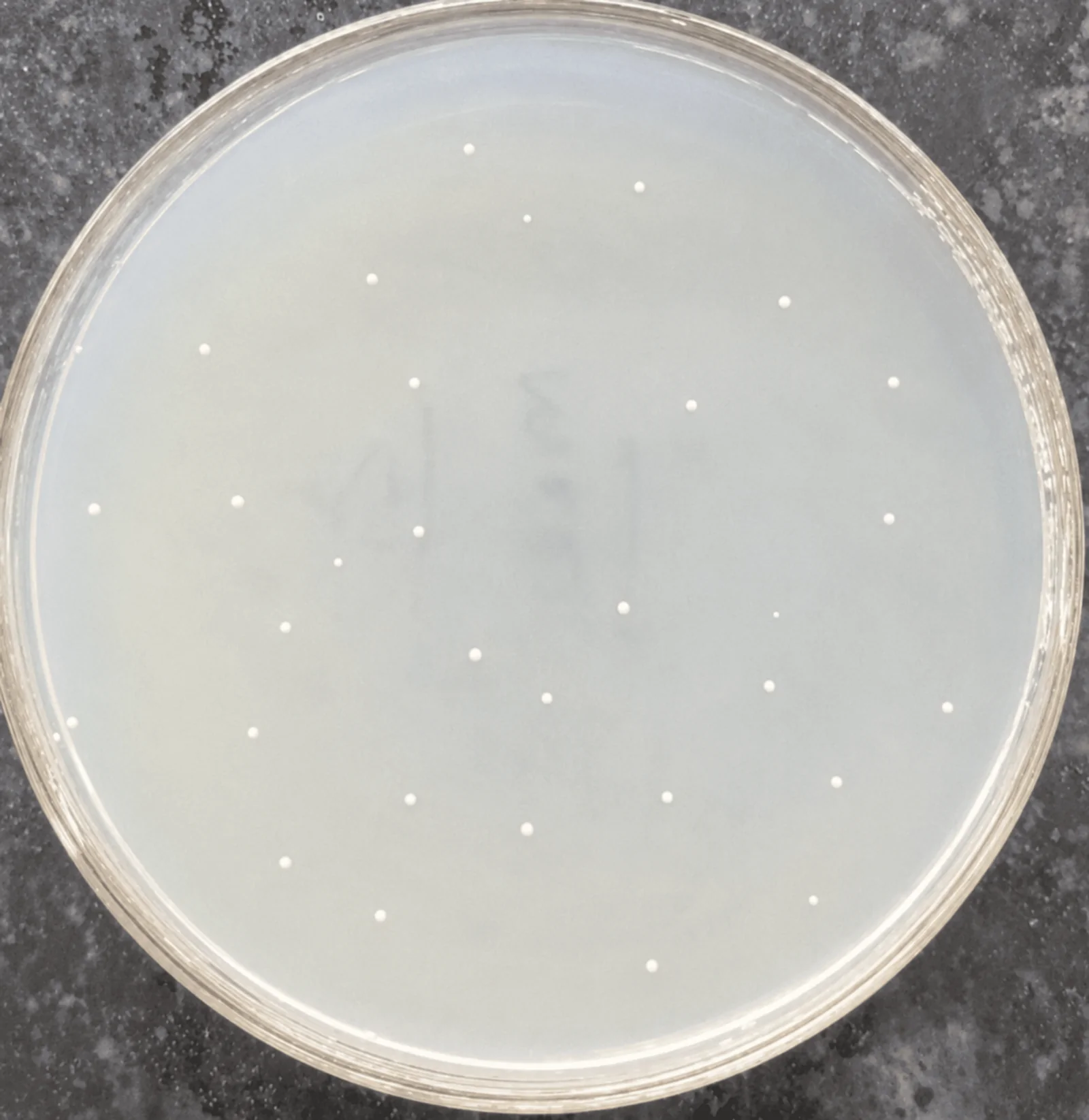
Two weeks postoperative oil pulling using virgin castor oil

Microbiological assessment

Unstimulated saliva samples were collected from each participant. MacConkey agar was prepared according to the manufacturer’s instructions. The saliva samples were serially diluted, and one loopful of the final dilution was streaked onto MacConkey agar plates, which were subsequently incubated aerobically at 37°C for 24 hours. After incubation, bacterial colonies were examined and counted manually using a colony counter. Colony counts were semi-quantified by multiplying by the dilution factor (1 × 10³), and the results were expressed as colony-forming units per millilitre (CFU/mL) of saliva. Monitoring longitudinal shifts in the oral bacterial composition is critical when evaluating the therapeutic efficacy of oral health interventions [30].

Statistical analysis

Data were entered into a spreadsheet and analysed using appropriate statistical software. The normality of the PI, GI, and bacterial load data was assessed using the Shapiro-Wilk test. Since the data satisfied the assumption of normality, parametric tests were employed. One-way ANOVA was used to compare the mean values among the three groups at each time point. Where a statistically significant difference was detected by ANOVA, Tukey’s Honest Significant Difference (HSD) post-hoc test was applied to identify specific pairwise differences between groups. The level of statistical significance was set at p < 0.05.

## Results

Group B exhibited the most pronounced decrease in PI scores (3.20 ± 0.40) (Table 1). At baseline, PI differed significantly among groups (p = 0.018), with means of 3.50 ± 0.44, 3.20 ± 0.40, and 3.65 ± 0.48 for Groups A, B, and C, respectively. At two weeks, the corresponding means were 2.90 ± 0.42, 2.50 ± 0.38, and 3.20 ± 0.44 (p < 0.001). Descriptive changes were -0.60, -0.70, and -0.45.

**Table 1:** Comparison of mean Plaque Index (Turesky modification of Quigley-Hein) scores among the three groups at baseline and two-week post-intervention assessment *Statistically significant (p < 0.05) SD: standard deviation; ANOVA: analysis of variance

Time Point	Group A (Coconut Oil) Mean ± SD	Group B (Castor Oil) Mean ± SD	Group C (Control) Mean ± SD	ANOVA F-value	p-value
Baseline (T0)	3.50 ± 0.44	3.20 ± 0.40	3.65 ± 0.48	F(2,42) = 4.05	0.018*
Postoperative (T2)	2.90 ± 0.42	2.50 ± 0.38	3.20 ± 0.44	F(2,42) = 10.79	<0.001*

The same trend can be observed when analyzing the GI values (Table 2). Two weeks after the intervention, a highly significant intergroup difference was observed (p < 0.001). In particular, Group B recorded the greatest improvement (0.90 ± 0.28), followed by Group A (1.00 ± 0.28), whereas Group C showed no meaningful change (1.80 ± 0.34). Thus, Group C showed minimal improvement in gingival condition.

**Table 2:** Comparison of mean Gingival Index (Löe and Silness) scores among the three groups at baseline and postoperative evaluations *Statistically significant (p < 0.05) SD: standard deviation; ANOVA: analysis of variance

Time Point	Group A (Coconut Oil) Mean ± SD	Group B (Castor Oil) Mean ± SD	Group C (Control) Mean ± SD	ANOVA F-value	p-value
Baseline (T0)	1.40 ± 0.30	1.50 ± 0.34	1.80 ± 0.36	F(2,42) = 5.82	0.009*
Postoperative (T2)	1.00 ± 0.28	0.90 ± 0.28	1.80 ± 0.34	F(2,42) = 40.20	<0.001*

Groups A and B showed a slight reduction in values, while Group C showed almost no change during the experiment (Table 3). By the second week after the intervention, a statistically significant reduction in bacterial load was observed in Groups A and B (5.70 ± 0.40 and 5.65 ± 0.38, respectively), compared with the control group (6.50 ± 0.38; p < 0.001), which had an initial value of 6.55 × 10³ CFU/mL.

**Table 3:** Comparison of mean salivary bacterial load (×10³ CFU/mL) among the three groups at baseline and postoperative evaluations *Statistically significant (p < 0.05) SD: standard deviation; CFU/mL: colony-forming units per millilitre; ANOVA: analysis of variance

Time Point	Group A (Coconut Oil) Mean ± SD	Group B (Castor Oil) Mean ± SD	Group C (Control) Mean ± SD	ANOVA F-value	p-value
Baseline (T0)	6.50 ± 0.36	6.35 ± 0.34	6.50 ± 0.40	F(2,42) = 0.83	0.461
Postoperative (T2)	5.70 ± 0.40	5.65 ± 0.38	6.50 ± 0.38	F(2,42) = 22.81	<0.001*

Table 4 presents the values of Δ for the three parameters analyzed. Group B (castor oil) had the highest Δ value, nearly equaling that of coconut oil (Group A). Group C (distilled water) did not show a comparable change.

**Table 4:** Descriptive mean changes in clinical and microbiological outcomes from baseline (T0) to two weeks (T2) Δ=T2-T0; negative values indicate lower follow-up values

Outcome	Group A (Δ T2-T0)	Group B (Δ T2-T0)	Group C (Δ T2-T0)
Plaque Index	-0.60	-0.70	-0.45
Gingival Index	-0.40	-0.60	0.00
MacConkey-culturable count (×10³ CFU/mL)	-0.80	-0.70	0.00

## Discussion

In addition to the reduction in PI and GI, the two-week regimen showed a reduction in the mean number of MacConkey-culturable bacteria in both the VCO and castor-oil groups relative to the distilled-water control group. Despite baseline differences in the mean PI and GI, the lack of adjustment for baseline values and the absence of a repeated-measures analysis in this trial constrained any inferential interpretation of the results. Although the castor-oil group had lower follow-up values for PI and GI compared with the VCO group, these descriptive changes did not demonstrate the superiority of castor oil.

OP is a complementary approach to standard hygiene practices, which include tooth brushing, flossing, and dental care [5,6]. In previous clinical investigations, the potential use of oils for oral malodor and gingivitis was reported. However, differences in oils, comparators, study populations, endpoints, and design features precluded direct comparability [7,8]. Reviews of coconut oil and holistic approaches to maintaining good oral health have also found biological plausibility but stressed the importance of using these approaches together with oral hygiene practices [9,10].

Differences in the types of studies investigating this issue are illustrated by a pilot trial among adolescents comparing the effects of sesame oil, ozonated sesame oil, and chlorhexidine [11]. Additionally, variable levels of awareness of OP among dentists and dental students demonstrated an incomplete evidence base [12]. Other studies have focused on OP and orthodontic material properties, radiation-induced mucositis, halitosis among orthodontic patients, multispecies biofilms, and periodontal and systemic inflammation [13-17]. Although these investigations indicate interest in oil-based interventions for oral health, they used quite different populations and endpoints and therefore cannot inform the effectiveness of VCO and/or castor-OP in otherwise healthy children.

Studies among the pediatric population have shown changes in caries activity and *S. mutans* counts after oil-pulling intervention [18]. Clinical trials have indicated short-term benefits from using VCO and other oils with respect to microbiological or gingival improvement [19,20], while another study performed among schoolchildren found less plaque accumulation and gingival inflammation after OP compared with other mouth-rinse strategies [21]. Although these changes were consistent with the current descriptive results, differences in age, baseline oral health, comparator interventions, measurements, intervention duration, and microbiological methods do not allow direct comparisons.

Some studies have found evidence for OP with respect to specific conditions, including oral malodor and xerostomia due to drug intake [22,23]. In addition, reviews of randomized trials and herbal interventions for plaque control identified potential short-term benefits from using OP in terms of oral health but found small samples, heterogeneous designs, methodological limitations, and poor evidence quality [24,25]. These findings indicate that the results of this study should be interpreted within the context of this limited evidence base rather than as confirmation of efficacy.

Due to the growing interest in OP, information on the Internet varies in terms of quality and completeness of content [26]. Similarly, a systematic review of traditional oral hygiene practices found promising practices but highlighted the need for rigorous validation before making recommendations for the general population [27]. These studies underline the need to discuss the preliminary nature of the evidence and discourage claims of equivalence or superiority of any oil-pulling method.

The mechanisms of action were not explored in this study. An in vitro study found that mechanical swishing, emulsification, and saponification might lead to more effective removal of bacteria and plaque from teeth [28]. Additionally, natural plant extracts and herbal agents have increasingly demonstrated notable antimicrobial efficacy against oral microbial species, supporting their role as natural oral hygiene supplements [29].

From T1 to T2, the descriptive decreases in PI and GI were 0.70 and 0.60, respectively, for the castor-oil group and 0.60 and 0.40, respectively, for the VCO group. The baseline-adjusted direct comparison of these small changes did not demonstrate the superiority of castor oil. The MacConkey-culturable bacterial counts descriptively decreased by 0.80 × 10³ CFU/mL in the VCO group and by 0.70 × 10³ CFU/mL in the castor-oil group, while the mean value in the control group remained constant. These microbiological changes were associated with the cultivation of certain organisms but cannot reflect changes in the overall oral microbiome.

No safety evaluation was carried out; consequently, the absence of adverse events cannot confirm the safety of either intervention. Following recommendations on the use of OP as a complementary practice [5,6], it should be emphasized that pediatricians should focus on gentle swishing, expectoration, avoiding swallowing or aspiration of the liquid, and adherence to other existing oral hygiene interventions.

The principal strengths of this study were the three-arm randomized design, inclusion of a distilled-water control, use of established PI and GI measures, and concurrent clinical and culture-based assessments. Nevertheless, the final sample was small (n = 15 per group), no a priori sample-size calculation was performed, and follow-up was limited to two weeks. Sequence generation and allocation concealment were insufficiently documented; examiner calibration and blinded outcome assessment were not reported. Significant baseline differences in PI and GI persisted despite randomization, while the one-way ANOVA approach did not account for within-participant correlation or adjust follow-up values for baseline.

Attrition was handled through complete-case analysis rather than intention-to-treat analysis. Adherence was supported by WhatsApp reminders but was not independently quantified. MacConkey agar measured a restricted Gram-negative culturable fraction and did not characterize total oral bacteria or key cariogenic and periodontopathic species; the plated volume, replicate procedure, and species identification were also incompletely documented. Castor oil’s viscosity and unpleasant taste could reduce acceptability and compliance in children, but these outcomes were not formally measured. Adverse events, aspiration symptoms, gastrointestinal effects, and acceptability were not systematically recorded. These limitations reduced reproducibility, generalizability, and confidence in comparative efficacy. Larger trials should use concealed randomization, calibrated blinded assessment, quantified adherence, baseline-adjusted mixed-effects or analysis of covariance (ANCOVA) models, intention-to-treat analysis, longer follow-up, and species-specific culture or molecular microbiome methods.

## Conclusions

In this trial, both VCO and castor-OP were associated with lower mean plaque and gingival-index scores and lower salivary MacConkey-culturable bacterial counts after two weeks than distilled-water rinsing. Because PI and GI differed at baseline and the analysis was not adjusted for baseline values or repeated observations, comparative treatment effects and castor-oil superiority were not established. MacConkey agar findings could not be generalized to total oral bacterial or cariogenic-pathogen reduction. Safety, acceptability, adherence, and cost-effectiveness were not formally evaluated; therefore, routine paediatric recommendation would be premature pending larger, methodologically rigorous trials.
